# Mitochondria in photosynthetic cells: Coordinating redox control and energy balance

**DOI:** 10.1093/plphys/kiac541

**Published:** 2022-11-28

**Authors:** Abir U Igamberdiev, Natalia V Bykova

**Affiliations:** Department of Biology, Memorial University of Newfoundland, St. John's, NL A1C 5S7, Canada; Morden Research and Development Centre, Agriculture and Agri-Food Canada, Morden, MB R6M 1Y5, Canada

## Abstract

In photosynthetic tissues in the light, the function of energy production is associated primarily with chloroplasts, while mitochondrial metabolism adjusts to balance ATP supply, regulate the reduction level of pyridine nucleotides, and optimize major metabolic fluxes. The tricarboxylic acid cycle in the light transforms into a noncyclic open structure (hemicycle) maintained primarily by the influx of malate and the export of citrate to the cytosol. The exchange of malate and citrate forms the basis of feeding redox energy from the chloroplast into the cytosolic pathways. This supports the level of NADPH in different compartments, contributes to the biosynthesis of amino acids, and drives secondary metabolism via a supply of substrates for 2-oxoglutarate-dependent dioxygenase and for cytochrome P450-catalyzed monooxygenase reactions. This results in the maintenance of redox and energy balance in photosynthetic plant cells and in the formation of numerous bioactive compounds specific to any particular plant species. The noncoupled mitochondrial respiration operates in coordination with the malate and citrate valves and supports intensive fluxes of respiration and photorespiration. The metabolic system of plants has features associated with the remarkable metabolic plasticity of mitochondria that permit the use of energy accumulated during photosynthesis in a way that all anabolic and catabolic pathways become optimized and coordinated.

## Teaser

Respiration efficiently coordinates redox and energy balance in photosynthetic cells through the change of the function of a mitochondrion from the powerhouse to the thermodynamic buffering organelle.

## Introduction

In plants, photosynthesizing cells possess two major systems for energy production associated correspondingly with chloroplasts and mitochondria. In the light, the primary energy-generating organelle is the chloroplast, while the physiological functions of mitochondria are modified compared to that of heterotrophic cells. In this review, we will discuss recent progress in understanding the role of mitochondria in photosynthetic metabolism and outlining their functions in photosynthetic plant cells.

The living state is characterized by the ability to prevail against disturbance through the system of self-maintaining resilient reaction networks (Heylighen et al., 2022). Metabolic organization underlies the structure of energy flows in a way that the useful energy transformation becomes maximized via the constrained release of energy that delays the production of entropy (Kauffman, 2020; Igamberdiev 2021). This means that energy flows in biological evolution can be evaluated via such economic criteria as productivity, efficiency, and the costs and benefits (profitability) of various mechanisms for capturing and utilizing energy to build biomass and do work (Corning, 2022), i.e. via the ability to prevail against disturbance, which is defined as ascendency (Ulanowicz, 1997, 2021).

**Figure 1:**
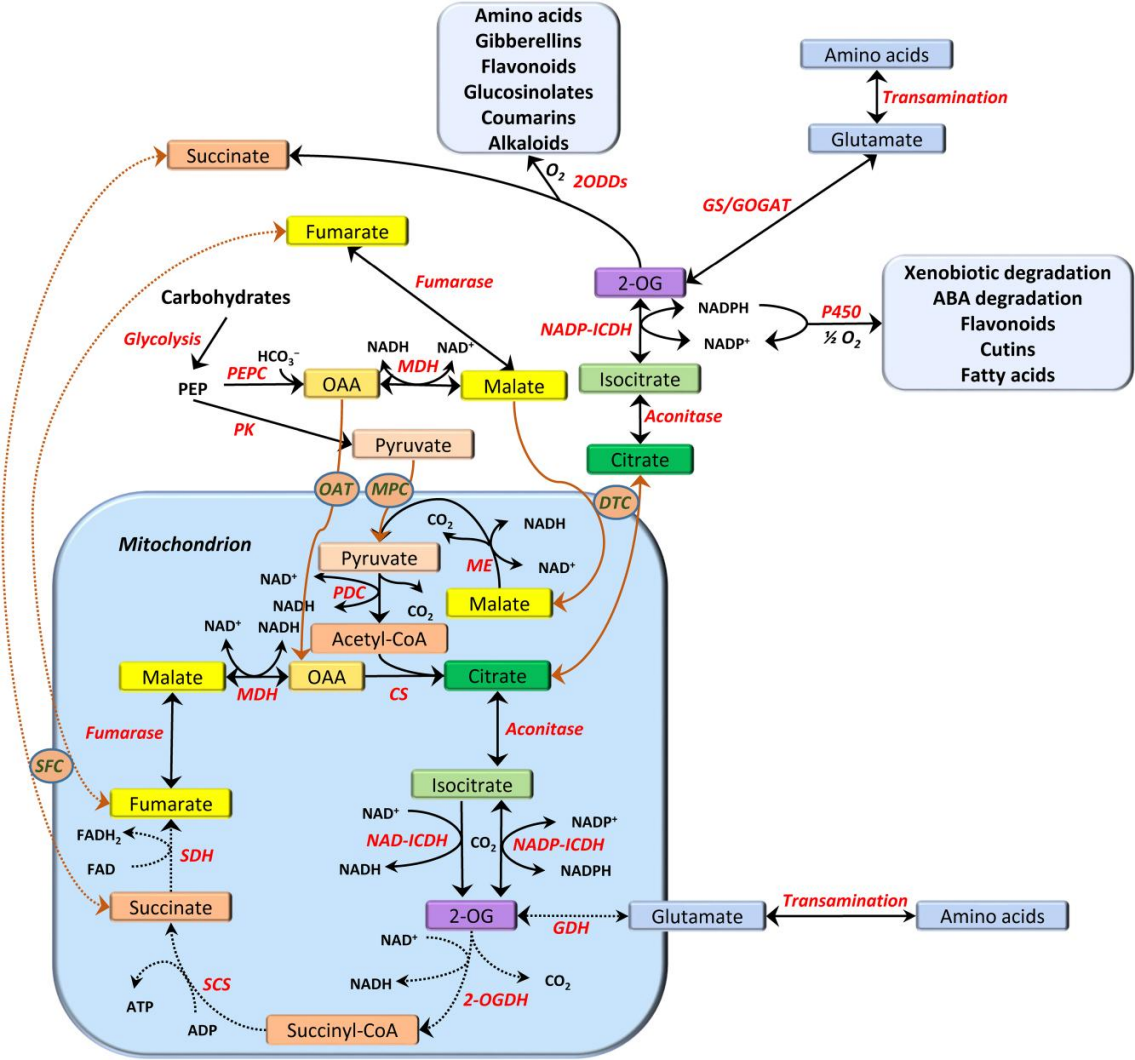
The open/closed modes of the TCA cycle using malate, oxaloacetate (OAA), or pyruvate as substrates and exporting citrate. Citrate conversion in the cytosol results in the formation of NADPH, which fuels cytochrome P450 monooxygenase reactions, and 2-OG, which can be dioxygenated, and both processes result in the formation of numerous secondary compounds. 2-OG can also be transaminated to glutamate, which leads to the biosynthesis of different amino acids. Abbreviations of enzymes: CS, citrate synthase; GC/GOGAT, glutamine synthetase/glutamate synthase; GDH, glutamate dehydrogenase. NAD-ICDH, NAD-dependent isocitrate dehydrogenase; NADP-ICDH, NADP-dependent isocitrate dehydrogenase; MDH, NAD-malate dehydrogenase; ME, NAD-malic enzyme; 2ODDs, 2-oxoglutarate-dependent dioxygenases; 2-OGDH, 2-oxoglutarate dehydrogenase complex; PDC, pyruvate dehydrogenase complex; PEPC, phosphoenolpyruvate carboxylase; PK, pyruvate kinase; SCS, succinyl-CoA synthetase; SDH, succinate dehydrogenase; Abbreviations of transporters: OAT, oxaloacetate transporter; MPC, mitochondrial pyruvate carrier; DTC, dicarboxylate-tricarboxylate carrier; SFC, succinate–fumarate carrier. The processes operating actively in the light are shown by the solid lines, whereas the dotted lines indicate processes suppressed in the light. The black lines correspond to biochemical reactions, and the brown lines designate the transport of compounds across membranes.

The metabolic system of plants possesses unique features that permit the use of energy accumulated in photosynthesis in a way that all anabolic and catabolic pathways become optimized and coordinated. Mitochondria are directly involved in this role in photosynthetic tissues via catalyzing the exchange of malate and citrate, contributing to the balance of adenylates, NAD, and NADP in the cytosol and other compartments, and via driving the operation of the cytochrome P450 and 2-oxoglutarate-dependent dioxygenase (2ODD) systems. The latter systems generate a vast number of secondary metabolites and other compounds determining the specificity and metabolic uniqueness of individual plant species (Figure 1).

Mitochondrial respiration is an important factor providing metabolic pathway flexibility by regulating the metabolic fluxes of supply and demand, and adjusting enzyme capacities (O’Leary et al., 2019, O’Leary, 2021). Respiration in the light provides an efficient mechanism for controlling redox and energy balance, and mitochondria change their role from being the energy powerhouse in the absence of photosynthesis to the thermodynamic buffering organelle in actively photosynthesizing cells (Igamberdiev and Kleczkowski, 2019). In the last years, a substantial advancement in our understanding of the role of mitochondria during photosynthesis has been achieved, but several important questions remain unresolved. Their resolution has important practical implications for the development of advanced engineering strategies involving plant respiration. New approaches will aim to slow down the unnecessary protein turnover, replace, relocate, or reschedule metabolic activities, suppress futile cycles, make ion transport more efficient, and thus boost plant productivity (Amthor et al., 2019). In this article, we will focus on the peculiarities of plant respiratory metabolism, its organization, and intercompartmental arrangement during photosynthesis to define the roles of respiration and mitochondria in the process of photosynthesis.

## Malate valve supplies the substrate for plant mitochondria

Since chloroplast membranes are almost impermeable to large charged molecules such as NADPH and ATP, the shuttle systems are essential for exchanging redox equivalents and energy between chloroplasts and cytosol. The malate valve is involved in shuttling redox equivalents across the inner chloroplast envelope. The reducing power is generated in the light reactions of chloroplasts as NADPH and is delivered to the cytosol, mitochondria, and other compartments in the form of NADH, which is formed via the reactions catalyzed by multiple isoforms of malate dehydrogenase (MDH) present in several plant cell organelles (Liszka et al., 2019; Selinski and Scheibe, 2019). In animal mitochondria, the oxidation of NADH drives ATP generation in oxidative phosphorylation, while in plant mitochondria, NADH oxidation may be uncoupled from ATP synthesis. Contrary to animal mitochondria, plant mitochondria can also oxidize NADPH, so the turnover of NADP constitutes an important feature of plant mitochondrial metabolism (Møller and Rasmusson, 1998; Møller et al., 2021; Figure 1; Box 1).

Box 1 **TCA cycle in an open mode**

In heterotrophic cells and in darkness, mitochondria operate as the main bioenergetic power stations, while during photosynthesis, this function is transferred to chloroplasts. In the light, mitochondria coordinate the phosphorylation of adenylates and the reduction of pyridine nucleotides in the cytosol and other compartments to avoid overenergization and to optimize major metabolic fluxes. This function, which can be defined as thermodynamic buffering, is achieved via the establishment and regulation of local equilibria of the TCA cycle enzymes. In the dicarboxylic branch of the TCA cycle, it is associated mainly with MDH and fumarase, while in the tricarboxylic branch, it is linked with aconitase and NAD- and NADP-dependent isocitrate dehydrogenases. When the redox level is elevated, the TCA cycle is transformed into a noncyclic open structure (hemicycle), leading to the export of the TCA (mainly citrate) to the cytosol and to accumulation of dicarboxylic acids (malate and fumarate). While the buildup of NADPH in chloroplasts supports the operation of the malate valve leading to the establishment of NADH/NAD^+^ ratios in different cell compartments, the production of NADH by mitochondria drives the export of citrate by establishing conditions for the operation of the citrate valve. The latter regulates the intercompartmental NADPH/NADP^+^ ratios, contributes to amino acid biosynthesis, and supports various reactions of secondary metabolism. Citrate exported from mitochondria stimulates the expression of AOX, thus facilitating metabolic turnover during active photosynthesis. The open mode of the TCA cycle and the activation of the noncoupled pathways of electron transport in the light are the main roles of mitochondria during photosynthesis, maintaining the organization and energy flow structure of the system and optimizing useful energy transformation (Figure 1).

In a recent study, Moreno-García et al. (2022) showed that suppression of the malate valve increases the NADH/NAD^+^ ratio in the cytosol, confirming the role of malate flux in the interaction between chloroplasts and the extrachloroplast part of the plant cell during photosynthesis. The Kok effect represents a phenomenon of changing abruptly the quantum yield of photosynthesis at low light and is often considered evidence for the suppression of respiration during photosynthesis (Yin et al., 2020). It is related to malate metabolism, which participates in maintaining the photosynthetic linear electron flow (Gauthier et al., 2020). The malate valve operates already during the stage of photosynthetic induction and is involved in the transfer of excess reducing power from chloroplasts via mitochondria, as the suppression of the mitochondrial NADH oxidation delays the start of photosynthesis (Igamberdiev et al., 1998, 2001). In the opposite process of the transition from light to darkness, defined as light-enhanced dark respiration (LEDR), photosynthetic products are oxidized in mitochondria in the form of malate, and the activation of malic enzyme is part of this process (Igamberdiev et al., 2001; Tronconi et al., 2015).

In C_4_ plants, malate provides CO_2_ for Rubisco in the bundle sheath cells. The NAD- and NADP-dependent malic enzymes are the part of malate exchange system between organelles, which acquires a new function in this particular group of plants. This function is attributed to the second isoform of NAD-malic enzyme of NAD-malic enzyme C_4_ species, in addition to the isoform that continues to fulfill respiratory functions (Hüdig et al., 2022). In the origin and evolution of C_4_ plants, mitochondria played a crucial role (Fan et al., 2022a), which resulted in a change in their metabolic function in NAD-malic enzyme and phosphoenolpyruvate kinase types of C_4_ plants. This was accompanied by an increased mitochondrial abundance and size, enzymatic capacity, and alterations in location and ultrastructure. At the same time, the level of dark respiration is not determined by mitochondrial capacity in C_4_ leaves, but it is primarily driven by cellular maintenance demands independently of mitochondrial organic acid cycling in the light (Fan et al., 2022b).

A diel flux balance analysis model by Shameer et al. (2019) substantiates the necessity of the dynamic mitochondrial respiration for the leaf energy balance in the light. Although chloroplasts can generate sufficient ATP to satisfy the energy requirements of the rest of the cell, the availability of chloroplast-derived ATP is limited by chloroplast energy dissipation, e.g. nonphotochemical quenching, and by the capacity of the chloroplast ATP export shuttles. The model shows that the chloroplast malate valve together with the triose phosphate-3-phosphoglycerate shuttle has an important metabolic role in maintaining the leaf energy balance during photosynthesis. The chloroplast malate valve complements the cyclic electron transport and chlororespiration associated with the NAD(P)H-dehydrogenase pathway to prevent over-reduction of the chloroplast (Chadee et al., 2021). However, instead of avoiding NADPH production or dissipating it, the malate valve directs the reductant to other cell compartments. When the noncoupled pathways in chloroplasts are suppressed by down-regulating their components, the expression of the mitochondrial alternative oxidase (AOX) increases, suggesting the complementary roles of these pathways for preventing over-reduction in photosynthetic cells (Chadee et al., 2021).

## The citrate valve generates the efflux of citrate and 2-OG as the biosynthetic products of plant mitochondria

**Figure 2:**
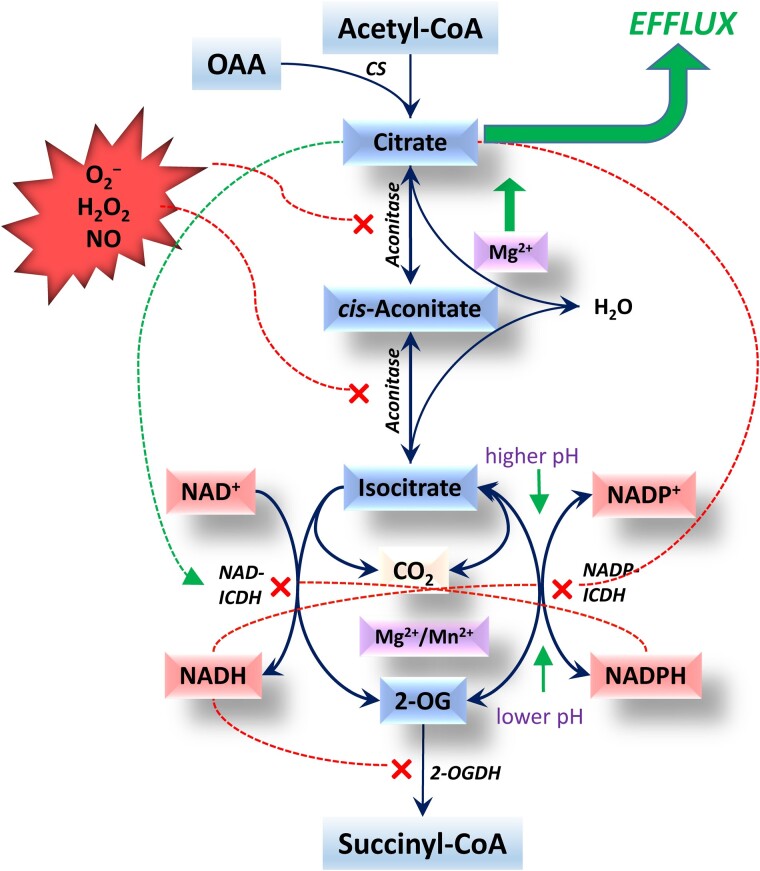
TCA branch of the TCA cycle and its regulation. Superoxide anion (O_2_^−^), hydrogen peroxide (H_2_O_2_), and nitric oxide (NO) inhibit aconitase via interaction with its iron–sulfur cluster. The irreversible NAD-dependent isocitrate dehydrogenase (NAD-ICDH) and the reversible NADP-dependent isocitrate dehydrogenase (NADP-ICDH) constitute the substrate cycle responding to the changes in redox levels of NAD and NADP. NADH inhibits both enzymes, NADPH inhibits NAD-ICDH and stimulates the reverse reaction of NADP-ICDH, and citrate stimulates NAD-ICDH and inhibits NADP-ICDH. Mg^2+^ displaces the equilibrium of aconitase toward citrate and activates both ICDHs (also Mn^2+^). The inhibition of aconitase and both ICDHs and stimulation of the reverse reaction of NADP-ICDH result in the efflux of citrate from mitochondria. CS, citrate synthase; OAA, oxaloacetate; 2-OG, 2-oxoglutarate; 2-OGDH, 2-oxoglutarate dehydrogenase complex. The blue lines indicate biochemical reactions, the red dotted lines indicate the enzyme inhibition, and the green dotted line shows the activation of NAD-ICDH by citrate.

The reducing power from chloroplasts is delivered to other cell compartments via malate in the form of NADH as NAD-MDH has only a low (about 1% of NAD^+^) affinity for NADP^+^ (Agius et al., 1998). However, numerous metabolic reactions, in particular biosynthetic reactions, require NADPH, and many of these reactions cannot be performed in chloroplasts. Thus, during active photosynthesis, there is a need for an engine that generates NADPH (Smith et al., 2021). The NADPH-generating systems include the oxidative pentose phosphate pathway and the system of NAD- and NADP-dependent glyceraldehyde-3-phosphate dehydrogenases (Wieloch, 2021). However, mitochondria play an important role in NADP turnover and NADPH generation (Figure 2; Box 2).

Box 2 **Aconitase equilibrium and isocitrate dehydrogenase redox cycle**

While citrate is formed by citrate synthase, which is activated in the light via reduced thioredoxin (Schmidtmann et al., 2014), its further conversion or efflux depends on the enzymes metabolizing it in the TCA cycle. Mg^2+^, the release of which takes place upon a decrease in ATP level, finely regulates the aconitase equilibrium by shifting it toward citrate (Igamberdiev and Kleczkowski, 2019), while ROS and RNS formed upon elevation of the cellular redox level inhibit aconitase and stimulate citrate efflux (reviewed by Igamberdiev and Bykova, 2018). The regulation of NAD- and NADP-dependent isocitrate dehydrogenases in mitochondria by the level of reduced and oxidized pyridine nucleotides is another point of fine regulation of citrate efflux/conversion. This pair of enzymes represents a system strongly responding to the intramitochondrial NADPH and NADH levels. Under high reduction levels of NADP and NAD, when CO_2_ is limited in the light, isocitrate oxidation in mitochondria is suppressed and citrate is transported to the cytosol, where the cytosolic NADP-ICDH supplies 2-OG for photorespiratory ammonia refixation and for the reactions of 2-OG-dependent dioxygenases. The NADPH formed in the NADP-dependent isocitrate dehydrogenase reactions in the cytosol and peroxisomes can fuel cytochrome P450 monooxygenase reactions. Under elevated intramitochondrial redox levels, the 2-OG dehydrogenase complex is inhibited, which stimulates citrate and 2-OG efflux from mitochondria. All these reactions are regulated via thioredoxin and other post-translational modifications. This results in the fine regulation of the tricarboxylic branch of the TCA cycle and thus the efflux of its intermediates (mainly citrate) to the cytosol that fuels glutamate biosynthesis, phytohormone production, and other numerous reactions of secondary metabolism (Figure 2).

Inside mitochondria, NADP turnover is mainly connected to the balance of redox level by the coupling of NAD- and NADP-dependent mitochondrial isocitrate dehydrogenases (Rasmusson and Møller, 1990), in which the NAD-dependent enzyme operates in one direction and the NADP-dependent enzyme operates in both directions (Igamberdiev and Gardeström, 2003). A substantial rate of NAD-malic enzyme with NADP^+^ represents another source of NADPH in the mitochondrial matrix (Maier et al., 2011). Inside mitochondria, NADPH is oxidized by the internal dehydrogenase NDC activated by Ca^2+^ (Møller and Rasmusson, 1998; Møller et al., 2021). The balance of the reduced and oxidized NAD and NADP represents an important redox engine for the switching of the tricarboxylic acid (TCA) cycle operation between the complete and partial modes (Sweetlove et al., 2010).

Besides the production and utilization of NADPH in mitochondria, even more important is the production of NADPH in the cytosol driven by mitochondria via the efflux of citrate via the citrate valve (Igamberdiev, 2020). This results in the maintenance of the cytosolic NADPH/NADP^+^ ratio in the light at a stable level (Igamberdiev and Gardeström, 2003) to drive different metabolic processes. The NADPH/NADP^+^ ratio is sustained at a value around 1.0 under different conditions of light and CO_2_ supply (Gardeström and Igamberdiev, 2016).

Lee et al. (2021) established that DICARBOXYLATE CARRIER 2 (DIC2) plays a central role in the mitochondrial malate–citrate exchange in Arabidopsis (*Arabidopsis thaliana*). DIC2 imports malate against citrate export, which is especially important for dark–light transitions. Among several dicarboxylate and tricarboxylate transporters, DIC2 is most effective in facilitating malate uptake and citrate export. The impairment of this process via DIC2 down-regulation resulted in slower growth, citrate accumulation inside mitochondria, an increase in the respiration rate, sugar depletion, and facilitation of peroxisomal citrate metabolism in darkness. Other organic acid transporters carrying out similar functions can partially compensate for the DIC2 function (Lee et al., 2021). Transporters of dicarboxylates (and amino acids) include the uncoupling proteins (UCP), which combine the dissipating function with the transport of metabolites such as glutamate, aspartate, and dicarboxylates (Monné et al., 2018).

**Figure 3:**
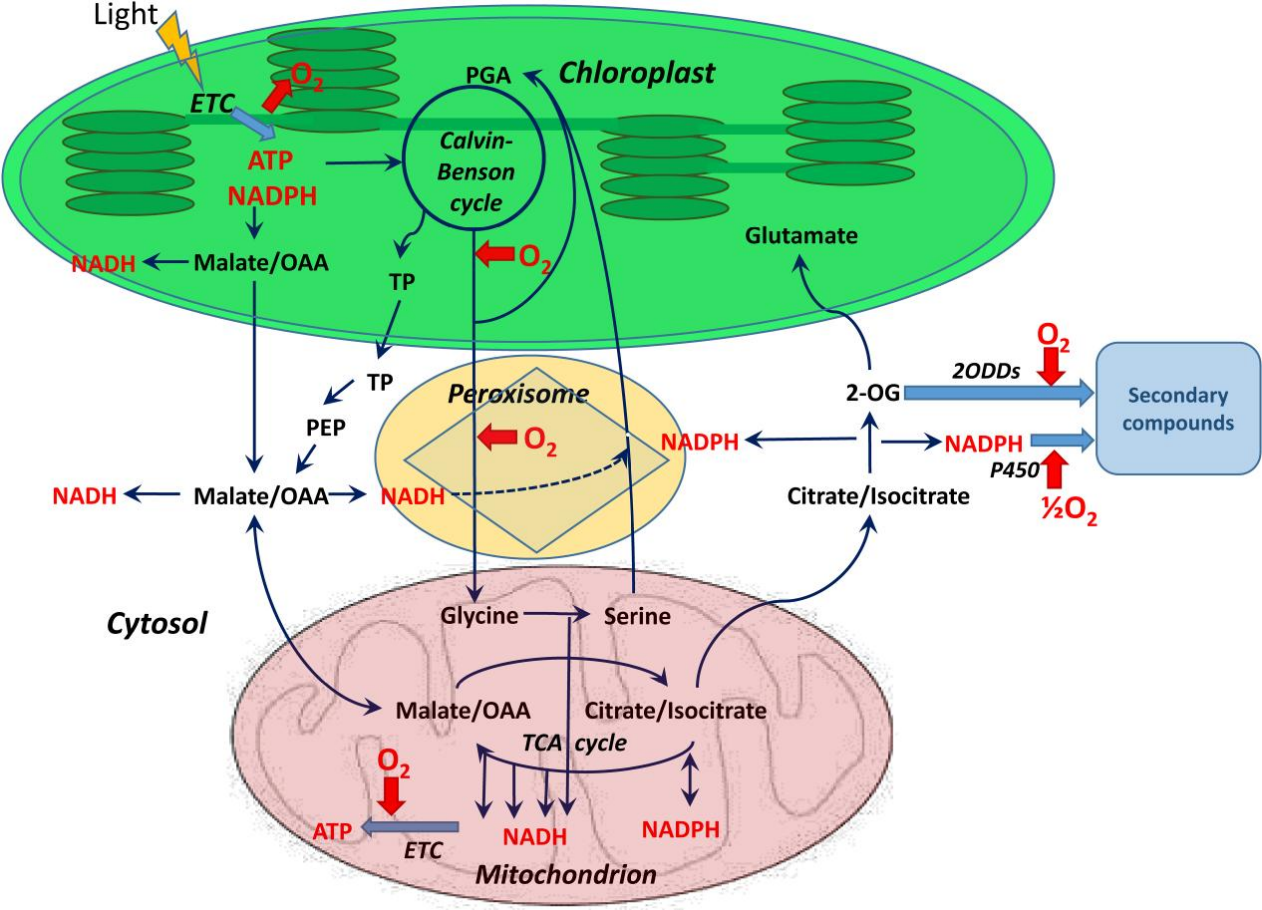
Metabolic coordination of chloroplasts and mitochondria in the light. NADPH formed in the photosynthetic ETC drives the Calvin–Benson cycle and fuels the malate valve in the reaction catalyzed by NADP-MDH. Oxidation of malate in the chloroplasts, cytosol, peroxisomes, and mitochondria by corresponding isoforms of NAD-MDH generates NADH in these compartments. In mitochondria, the increase of NADH level fuels the mitochondrial ETC and stimulates the efflux of citrate, which is transformed into 2-OG by the cytosolic aconitase and NADP-isocitrate dehydrogenase. The increase in the cytosolic NADPH level results in stimulation of the cytochrome P450-dependent monooxygenase reactions leading to the synthesis of secondary metabolites, xenobiotic degradation, and other processes. The rise in the 2-OG level results in glutamate synthesis and 2ODD reactions, which also generate secondary metabolites. Oxygen produced in photosynthesis is used not only for mitochondrial respiration but also in Rubisco oxygenase reaction (photorespiration), glycolate oxidase in peroxisomes, and in mono- and dioxygenase reactions, leading to the formation of secondary metabolites. OAA, oxaloacetate; PEP, phosphoenolpyruvate; PGA, 3-phosphoglyceric acid; TP, triose phosphate. The oxygen consumption or release is shown by the red arrows, and the light blue thick arrows indicate the formation of secondary compounds. The biochemical reactions and transport processes are represented by the solid thin lines, and the dotted line indicates the reduction of hydroxypyruvate in peroxisomes by NADH.

The biosynthetic reaction of citrate synthesis from malate via the TCA cycle has features that combine light–dark metabolism. While NAD-malic enzyme is more active in the dark, the mitochondrial citrate synthase (Eprintsev et al., 2018b) and pyruvate dehydrogenase complex (PDC; Tcherkez et al., 2005) are inhibited by light. The pyruvate imported via the pyruvate carrier forms a distinct pool independent of the pyruvate pools originating from NAD-malic enzyme and from the transamination of alanine (Le et al., 2022). The citrate efflux can be stimulated by the high redox level in the light (Igamberdiev, 2020). Citrate exhibits a fundamental structural role in the connectivity of the metabolic network serving as a link between different communities of enzymes and metabolites, and plays a central role in the integration of carbon and nitrogen metabolism in plants (Toubiana et al., 2016). A scheme showing interactions between mitochondria and chloroplasts in the light and the operation of malate and citrate valves is presented in Figure 3.

## Operation of malate and citrate valves during photorespiration

During photorespiration, an intensive flux of glycine through mitochondria of C_3_ plants occurs, which results in an increase in the intramitochondrial concentrations of NADH and NADPH, engaging components of the electron transport chain (ETC) internal dehydrogenases (NDA and NDC) and the alternative pathway associated with AOX (Bykova and Møller, 2001; Bykova et al., 2014). MDH in mitochondria participates in the oxidation of NADH, preventing substrate inhibition of glycine decarboxylase (Bykova et al., 2014; Lindén et al., 2016). While the engagement of the noncoupled pathways supports high photorespiratory flux, the photorespiratory ATP production remains high and supports biosynthetic reactions in the cytosol (Gardeström and Igamberdiev, 2016).

Malate/oxaloacetate equilibrium plays a key role in photorespiration not only for supporting glycine oxidation but also for hydroxypyruvate reduction in peroxisomes, where the malate valve supplies NADH for this reaction (Cousins et al., 2008; Dao et al., 2022). Photorespiration generates a large amount of NADH in mitochondria, exceeding its NADH-dissipating capacity, which leads to its export to the cytosol through the malate–oxaloacetate shuttle and to the maintenance of redox states of NADP and NAD pools in several subcellular compartments connected by the malate-OAA shuttles (Lim et al., 2020; Figure 3).

While the role of malate shuttling in photorespiration has a direct function in the intercompartmental redox transfer, the role of citrate efflux from mitochondria during photorespiration has received less attention. However, the operation of the citrate valve during photorespiration fulfills an important function for refixation of photorespiratory ammonia by supplying 2-oxoglutarate (2-OG) for glutamate biosynthesis (Fernie and Bauwe, 2020). Thus, by increasing the redox level in mitochondria, the photorespiratory flux transforms the TCA cycle into the open (hemicycle) mode and activates the citrate valve. This, in turn, stimulates the expression and activation of AOX by citrate, as well as activation of rotenone-insensitive dehydrogenases through the elevation of NADH/NAD^+^ ratio in mitochondria (Bykova et al., 2014).

Therefore, the operation of malate and citrate valves and the functioning of the TCA cycle in open mode (Figure 1; Box 1) represent prerequisites for the realization of high photorespiratory flux and for its preference over other respiratory reactions in C_3_ plants during photosynthesis. Although the flux through the TCA cycle is decreased under these conditions, in particular, due to the inhibition of PDC by photorespiratory ammonia and the inhibition of expression of several enzymes by light (reviewed by Igamberdiev and Bykova, 2018), the active intercompartmental exchange of malate and citrate is crucial for supporting high rates of photorespiratory flux. This is achieved by the prevention of glycine decarboxylase inhibition by NADH, by NADH-dependent hydroxypyruvate reduction (with the participation of MDH isoforms), via the activation of AOX transcription by citrate, up-regulation of the AOX activity by high redox and pyruvate levels, and switching to rotenone-insensitive dehydrogenases at high NADH and NADPH levels (Bykova et al., 2014).

## Switching to the noncoupled pathways in the light

Under conditions where ATP and redox power are intensively produced by light-dependent reactions of photosynthesis, chloroplasts have a very limited capacity to supply ATP to the cytosol. The redox power is supplied from chloroplasts to other cellular compartments in the form of malate, and the oxidation of malate by NAD-MDH generates NADH (Gardeström and Igamberdiev, 2016). Switching to the noncoupled pathways of mitochondrial electron transport, which balances ATP/ADP and NAD(P)H/NAD(P)^+^ ratios in mitochondria, cytosol, and other cell compartments, represents an important mechanism of achieving stable operation and maximum efficiency of photosynthetic plant cells. The mitochondrial capacity of supplying ATP and NADPH (generated primarily via isocitrate oxidation) remains an important precondition for cellular metabolism via the synergistic operation of different compartments in photosynthetic cells (Igamberdiev, 2020).

When the supply of ATP and redox equivalents to the cytosol by mitochondria is optimal, further energization of cytosol and mitochondria would negatively affect the efficient operation of photosynthetic cells. Equilibration of the fluxes of load and consumption of ATP and redox power in metabolic reactions is essential for a stable metabolic performance (Igamberdiev and Kleczkowski, 2019). Under these conditions, switching to the noncoupled pathways of electron transport plays an important role in the optimization of metabolic performance in photosynthetic cells.

Plant mitochondrial electron transport contains numerous components that are not coupled to the generation of proton potential and ATP synthesis (Møller et al., 2021; Popov et al., 2021). They include rotenone-insensitive dehydrogenases NDA, NDB, NDC, AOX, and UCP. The rotenone-insensitive dehydrogenases are encoded by seven genes in Arabidopsis and are responsible for four distinct activities: NDA oxidizes NADH at the internal side of the inner mitochondrial membrane, NDB1 oxidizes NADPH, and NDB2 oxidizes NADH externally, while NDC oxidizes NADPH internally (Møller et al., 2021). They are dual-targeted proteins, and the homologs of rotenone-insensitive dehydrogenases are present in peroxisomes and also in chloroplasts (Xu et al., 2013). The operation of NDB1 and NDC is Ca^2+^ dependent, and the NDB-type dehydrogenases operate at lower pH values (Rasmusson et al., 2008). A coordinated expression of the components of plant mitochondrial ETC has been shown, e.g. *AOX1a* and *AOX1d*, along with *NDB3*, are co-expressed during stress treatments in rice (*Oryza sativa)* (Wanniarachchi et al. 2018). In Arabidopsis, *NDB2a* and *AtAOX1A* are co-expressed (Sweetman et al. 2019). An integrated regulation of the chloroplast cyclic electron transport and the mitochondrial noncoupled pathways was also noted. A signal derived from the redox status of the photosynthetic ETC coordinately controls the amount of AOX and the light-harvesting complex protein LHCB2, and both proteins then contribute to the maintenance of chloroplast energy balance, particularly under stress conditions (Dahal et al., 2017; Chadee et al., 2021; Alber and Vanlerberghe, 2021).

**Figure 4:**
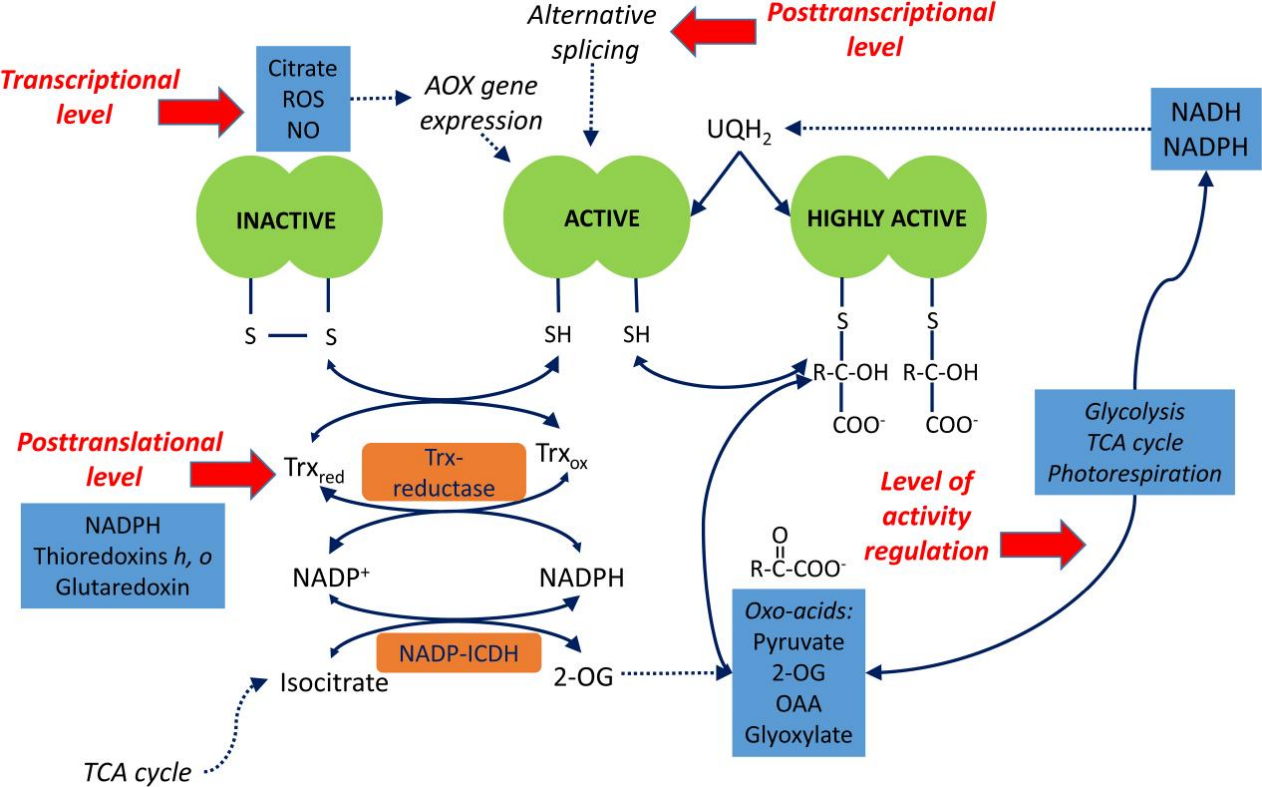
Multilevel regulation of AOX. AOX biosynthesis is regulated transcriptionally by citrate and by elevated levels of ROS and NO. AOX is modified post-translationally by the mitochondrial thioredoxins (Trx) *h* or *o*, which mediate the conversion between an inactive form with a disulfide bridge between the monomers and an active form with free thiol groups. Thioredoxin reductase regenerates Trx to the reduced form; NADPH for this reaction is supplied by NADP-dependent isocitrate dehydrogenase (NADP-ICDH). AOX is activated by oxo-acids including pyruvate (coming from the glycolysis and malic enzyme reactions), 2-OG and oxaloacetate (OAA; formed in the TCA cycle), and glyoxylate and hydroxypyruvate (from photorespiration). The post-translational binding of pyruvate and other oxo-acids to the reduced form further activates the enzyme. The increase in the concentration of the AOX substrate ubiquinol (UQH_2_) in the inner membrane stimulates AOX activity. The individual biochemical processes are shown with the solid arrows; and the dotted lines indicate regulatory effects. Thick red arrows define different levels of AOX regulation. The figure was substantially modified from Møller et al. (2020a) and the Web Figure 12.3C of Taiz et al. (2015): http://6e.plantphys.net/topic12.03.html.

Fine regulation of the noncoupled pathways takes place at all levels of molecular organization (Figure 4). In particular, at the genetic level, citrate activates the expression of the genes encoding the most abundant AOX1 form (but not AOX2), with isocitrate being the only organic acid to exhibit this effect (Finkemeier et al., 2013). The gene *NDA2* encoding the rotenone-insensitive internal NADH dehydrogenase with a higher *K*m(NADH) than complex I is also stimulated by citrate (Finkemeier et al., 2013). Thus, citrate triggers the transcription of the genes involved in the activation of the noncoupled pathways of mitochondrial electron transport. Citrate as a product of the TCA cycle becomes the main activator at the transcriptional level not only for AOX but also for NDA. Accumulation of ROS and NO contributes to the regulation of AOX at the transcriptional and post-translational levels (Florez-Sarasa et al., 2016; Ageeva-Kieferle et al., 2021). Light stimulates NDA and NDC expression (Escobar et al., 2004), and even stronger light-activated gene expression occurs for the external NDB dehydrogenases (Michalecka et al., 2003). Wallström et al. (2014) demonstrated that the down-regulation of NDB1 led to decreased levels of sugars, citric acid cycle intermediates, and amino acids, as well as to transcriptomic changes associated with protein synthesis, glucosinolate, and jasmonate metabolism. By using the mutants of two UCP, Arcuri et al. (2021) established that ROS homeostasis is altered at higher ATP/ADP ratios under the suppression of mitochondrial uncoupling.

At the post-translational level, AOX is regulated by the redox state via thioredoxin and by oxo-acids providing high flexibility of metabolism under changing environmental conditions (Florez-Sarasa et al., 2016). The specific mitochondrial thioredoxin, Trx *h*, is capable of activating AOX, e.g. the thioredoxin *h* from poplar (PtTrxh2) can be reduced efficiently by the mitochondrial Trx reductase AtNTRA, and then it can act as a reducing agent for AOX homodimers and facilitate AOX activation by pyruvate (Gelhaye et al., 2004; Figure 4). On the other hand, in pea (*Pisum sativum* L.), the activation of AOX was achieved by the *o*-type thioredoxin, which also interacted with other mitochondrial proteins, including peroxiredoxin and enzymes of the photorespiratory pathway (reviewed in da Fonseca-Pereira et al., 2021).

Initially, it was suggested that pyruvate is the main organic acid activator of AOX, and the function of this compound as the product of glycolysis and the entry of mitochondrial metabolism links AOX to the TCA cycle (Day et al., 1994). It was later demonstrated that different AOX isoforms have different sensitivity levels to pyruvate (Sweetman et al., 2022), and AOX isoforms are also differentially activated by the TCA cycle intermediates oxaloacetate (OAA) and 2-OG (Selinski et al., 2018). Thus, AOX possesses fine regulatory mechanisms for differential activation of isoforms by the intermediates of mitochondrial metabolism appearing at the entry (pyruvate), the citrate branch (2-OG), and the malate branch (OAA) of the TCA cycle. The activation of AOX by the photorespiratory intermediates glyoxylate and hydroxypyruvate (Pastore et al., 2001) reveals the metabolic adaptability of AOX toward the photorespiratory flux, which requires the oxidation of large amounts of NADH without coupling to the ATP synthesis (Igamberdiev et al., 1997; Bykova et al., 2014).

The dependency of stromal ATP production on the dissipation of photosynthetic reductants in mitochondria was shown in a study that used fluorescent probes to measure ATP in vivo (Voon et al., 2018). This study demonstrated that, during illumination, the provision and consumption of ATP/NADPH in chloroplasts can be balanced by exporting excess reductants rather than importing ATP from the cytosol. It is important to maintain the ATP/NADPH balance for the optimal photosynthetic performance, and the reductant dissipation in mitochondria plays the primary role in this process.

## Plant mitochondria are the drivers of 2ODD reactions

Plant mitochondria provide 2-OG for glutamate biosynthesis and through this supply intermediates for the biosynthesis of many other amino acids and their derivatives (Igamberdiev and Bykova, 2018). This function is the most evident consequence of the citrate valve (Igamberdiev, 2020). NADP-isocitrate dehydrogenase (NADP-ICDH) in the cytosol is a key player in 2-OG production that affects different aspects of metabolism including several secondary pathways (Araújo et al., 2014). The overexpression of NADP-ICDH causes alterations, resulting in enhanced plant growth and vascular development (Pascual et al. 2018). In mitochondria, 2-OG can be oxidized to succinate via the 2-OG dehydrogenase complex and succinyl-CoA synthetase coupled to ATP production. However, 2-OG can be converted to succinate in an alternative way through the reaction of its dioxygenation catalyzed by 2-OG-dependent dioxygenases (2ODDs). These reactions occur mostly in the cytosol and initiate several metabolic pathways leading to the formation of diverse physiologically important compounds including secondary metabolites (Islam et al., 2018; Figure 1; Box 3).

Box 3 **TCA cycle and secondary metabolism**

Through efflux of citrate from mitochondria, 2-OG production in the cytosol not only feeds glutamate biosynthesis but also supports a range of oxidative reactions catalyzed by 2-OG-dependent dioxygenases (Araújo et al., 2014). This drives glucosinolate, flavonoid, and alkaloid metabolism as well as gibberellic acid and amino acid metabolism. In particular, differences in the levels of bioactive gibberellin are controlled by the flux of 2-OG from the TCA cycle via 2-OG-dependent dioxygenases. The formation of 2-OG in the cytosol not only supports various 2-OG-dependent dioxygenase reactions but also produces NADPH to support cytochrome P450-driven monooxygenase reactions resulting in the formation of numerous secondary compounds (Figures 1 and 3). Understanding the regulation of monooxygenase and dioxygenase reactions via the TCA cycle opens great possibilities for bioengineering the production of particular secondary metabolites by plants.

2ODDs represent a large superfamily of enzymes with mostly Fe(II) as a redox cofactor, which use 2-OG and O_2_ as substrates and form CO_2_ and succinate as products. In the course of this reaction, various compounds are oxidized via hydroxylation, including small molecules as well as proteins, nucleic acids, and lipids. Furthermore, oxidative modifications catalyzed by 2ODDs include not only hydroxylations but also demethylations (that include *N*-methyl and, in some cases, *O*-methyl demethylations), desaturations, ring closure, ring cleavage, epimerization, rearrangement, halogenation, and demethylenation (Farrow and Facchini, 2014; Sonawane et al., 2022; Song et al., 2022). In primary metabolism, 2ODDs participate in DNA repair and histone modifications, epigenetics, post-translational modifications, hypoxia response, and activation and catabolism of plant growth regulators. In the specialized (secondary) metabolism, 2ODDs participate in numerous pathways and display as much functional diversity as cytochrome P450 monooxygenases (Araújo et al., 2014).

The TCA cycle modulates not only the flux from 2-OG to amino acid metabolism but also the flux through 2ODDs that connects the TCA cycle with glucosinolate, flavonoid, and alkaloid formation, as well as with different reactions of amino acid and GA biosynthesis (reviewed by Araújo et al., 2014). The direct role of the TCA cycle in these reactions was shown through the inhibition of the mitochondrial 2-OG dehydrogenase complex (Araújo et al., 2012a, 2012b; Condori-Apfata et al., 2021). In the mitochondrial matrix, citrate synthase controls not only the production of citrate that leads to the formation of 2-OG entering the 2ODD reactions but also the formation of products of metabolic pathways initiated by 2ODDs, e.g. of anthocyanin synthesis in petunia (Zhao et al., 2021). Some genes encoding ODDs are activated by light (Cho et al., 2012).

The level of 2-OG in mitochondria and cytosol is balanced via its oxidation in 2-OG dehydrogenase reaction, dioxygenation by 2ODDs, amination to glutamate, and other reactions. Its concentration directly regulates pyruvate kinase and PEP carboxylase in the cytosol, and citrate synthase and AOX in mitochondria (Araújo et al., 2014). These enzymes, in turn, control the fluxes through glycolysis and the TCA cycle, thus establishing an autocatalytic feedback mechanism in the metabolic system that leads to its balanced operation.

## Plant mitochondria energize cytochrome P450 monooxygenase reactions

The formation of 2-OG in the cytosol not only supports various 2ODD reactions but also produces NADPH that can deliver electrons to cytochrome P450-driven monooxygenase reactions. The latter result in the formation of various secondary compounds and bioactive products, of which there are over 200,000 identified substances (Jensen and Møller, 2010; Jensen et al., 2011; Pandian et al., 2020; Hansen et al., 2021). An altered cellular redox status in mitochondria was shown to be a key factor in the coordination of proline and very long-chain fatty acid metabolism through the involvement of cytochrome P450 (Shinde et al., 2016). In cyanobacteria, the expression of one cytochrome P450 protein (CYP1A1) results in the reduction in the expression of other natural electron dissipation pathways, and cytochrome P450 can be used as a competing electron sink (Torrado et al., 2022).

Thus, 2-OG-dependent dioxygenases and cytochrome P450 monooxygenases not only utilize oxygen for the formation of numerous bioactive compounds but also their operation is driven by mitochondria supplying 2-OG and NADPH via the citrate valve (Figure 1; Box 3). Further experimental work is needed to clarify the direct role of mitochondria in fueling secondary metabolism via the monooxygenase and dioxygenase reactions.

## Multilevel regulation of respiration in the light

**Figure 5:**
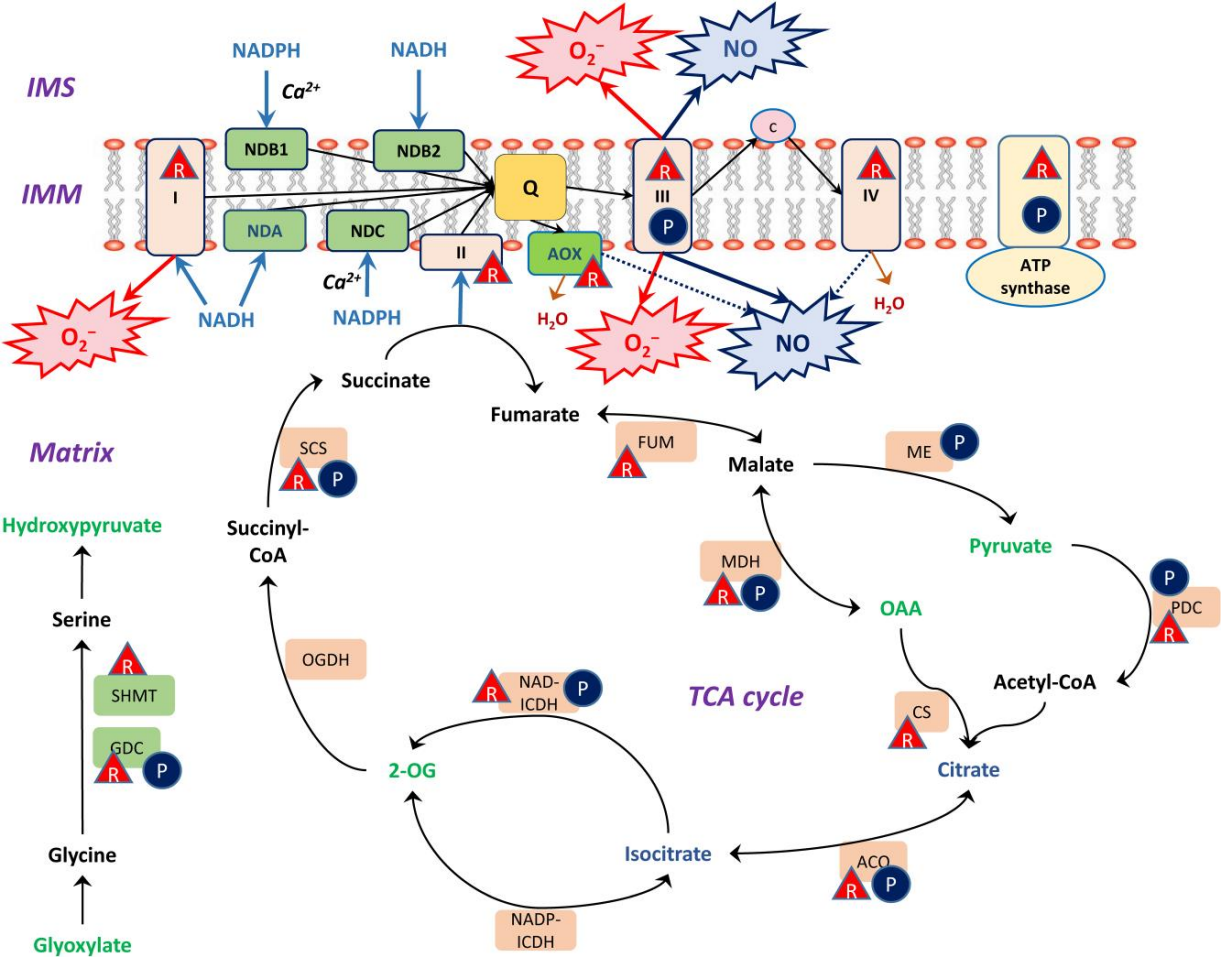
Redox and phosphorylation post-translational modifications and the regulation of TCA cycle enzymes and ETC components. Phosphorylation is shown by circled P, and Trx-dependent reduction is depicted by triangles with R. Abbreviations of the mitochondrial matrix enzymes and ETC components: ACO, aconitase; AOX, alternative oxidase; I, II, III, IV, complexes I–IV; CS, citrate synthase; FUM, fumarase; GDC, glycine decarboxylase complex; ICDH, isocitrate dehydrogenase; IMM, inner mitochondrial membrane; IMS, intermembrane space; MDH, malate dehydrogenase; NDA, internal rotenone-insensitive NADH dehydrogenase; NDB1 and NDB2, external rotenone-insensitive NADPH and NADH dehydrogenases; NDC, internal rotenone-insensitive NADPH dehydrogenase; OGDH, 2-oxoglutarate dehydrogenase complex; PDC, pyruvate dehydrogenase complex; SCS, succinyl-CoA synthetase; SHMT, Ser hydroxymethyltransferase. Oxo-acids activating AOX isoforms are highlighted in green; citrate and isocitrate causing transcriptional activation of AOX and NDA are highlighted in blue. The biochemical reactions and electron transport are indicated by black thin lines with arrows, whereas H_2_O formation is shown in brown color. The light blue arrows indicate the supply of redox equivalents to ETC, the dark blue arrows indicate nitric oxide (NO) production, and the red arrows show superoxide anion (O_2_^−^) production. The figure was substantially modified from Møller et al. (2020a)

Regulation of respiration in the light represents a phenomenon that coordinates all major pathways of metabolism during photosynthesis including reactions of secondary metabolism. It occurs at all levels of molecular organization and includes transcriptional, post-transcriptional, and post-translational mechanisms. Covalent post-translational modifications of proteins represent an important mode of regulation associated with changes in redox and energy balance in the cell (Møller et al., 2020a, 2020b; Figure 5). Fine regulation mechanisms take place at the level of activity modulation of enzymes by metabolites and cofactors. A consequence of energy production in chloroplasts is its involvement in the regulation of transcription of the mitochondrial genome. A study recently showed that overexpressed dual-targeted purple acid phosphatase 2 (AtPAP2) participated in the import of several nuclear-encoded proteins into chloroplasts and mitochondria (Liang et al., 2015).

While AOX and NDA are activated by citrate at the transcription level (Finkemeier et al., 2013), the post-transcriptional mode of regulation via alternative splicing is essential. The role of alternative splicing in the expression of AOX was clearly demonstrated (Kong et al., 2003), as well as the expression of subunits of mitochondrial ETC complex I (Lee et al., 2017; Lin et al., 2022). Another major mechanism of regulation of mitochondrial proteins is the methylation of promoters, as shown in the case of NAD-malic enzyme, aconitase, and citrate synthase (Eprintsev et al., 2018b, 2018a, 2020a, 2020b, 2022). Succinate dehydrogenase and fumarase represent an important point of control of the TCA cycle in the light (Huang et al., 2019). Their regulation is achieved by phytochrome and cryptochrome at the transcriptional level via methylation of promoters (Eprintsev et al., 2018a) and via thioredoxin at the post-translational level (Daloso et al., 2015).

Since the maintenance of redox and energy balance denotes the main mechanism for achieving the stable operation of photosynthetic plant cells, sudden changes in the cellular environment trigger numerous signaling events that are related to the regulation at different levels. Redox signaling refers to the operation of the thioredoxin system and to the use of reactive oxygen and nitrogen species (ROS and RNS) as signaling compounds that initiate feedback mechanisms to keep the operation of photosynthetic cells under control. In mitochondria, ROS and RNS are formed upon elevation of the redox state during active photosynthesis, and the noncoupled pathways are involved in the regulation of their production and scavenging (Cvetkovska and Vanlerberghe, 2012; Hebelstrup and Møller, 2015; Van Aken, 2021). Over-reduction of mitochondria and ROS formation take place as a result of heat stress due to the increased fluidity of membranes and impairment of cytochrome c oxidase (Scafaro et al., 2021). Many redox-related reactions are mediated via the pool of glutathione and ascorbate. A highly negative glutathione redox potential (*E*_GSH_) is maintained in the cytosol, plastids, and mitochondria of plant cells to support fundamental processes, including antioxidant defense, redox regulation, and iron–sulfur cluster biogenesis (Marty et al., 2019). Glutathionylation of proteins may represent another important redox regulatory mechanism (Palmieri et al., 2010), although its contribution to interactions between photosynthesis and respiration requires further investigation (reviewed in Møller et al., 2020a, 2020b).

Although RNS is formed at high levels under low oxygen conditions when the components of electron transport use nitrite instead of oxygen as the terminal electron acceptor to produce NO, the activity of electron transport components toward generating NO also takes place under normoxic conditions in the light (Gupta et al., 2018). In normoxic conditions, the Q-cycle of complex III generates NO from nitrite, and AOX reduces this activity by acting as a nonenergy-conserving electron sink upstream of complex III (Cvetkovska and Vanlerberghe, 2012; Hebelstrup and Møller, 2015; Alber et al., 2017). The role of AOX in anaerobic conditions can be related to NO generation, while in normoxia, the AOX engagement contributes to NO scavenging (Jayawardhane et al., 2020). Recent evidence strongly indicates that NO signaling could play an important role during photosynthesis and photorespiration via redox protein modification, such as *S*-nitrosylation of glycine decarboxylase (Palmieri et al., 2010; Keech et al., 2017) and tyrosine nitration of superoxide dismutases by peroxynitrite formed via NO and superoxide interaction (Holzmeister et al., 2015).

An important role in the regulation of plant respiration during photosynthesis belongs to protein phosphorylation (Figure 5). The reversible phosphorylation of Ser, Thr, and Tyr residues in proteins represents a dynamic protein regulatory mechanism that operates in concert with changes in the ATP/ADP ratio in plant cells. By using either [γ-^32^P]ATP labeling-based or mass spectrometry-based phosphoproteomic analysis, more than 50 phosphorylated proteins and a number of protein kinases and protein phosphatases have been identified in isolated plant mitochondria, including several TCA cycle enzymes, ETC components, and components of most other major mitochondrial pathways (Bykova et al., 2003; Havelund et al., 2013; reviewed in Møller et al., 2020a, 2020b). Among these proteins, the regulation of only PDC is understood in detail (Tovar-Méndez et al., 2003). The activity of MDH decreases with an increase in the ATP/ADP ratio (Yoshida and Hisabori, 2016), and redox regulation by Trx was also observed (Martí et al., 2020). The phosphorylation cascades are especially important for triggering autophagy during cell senescence. This includes mitogen-activated protein kinase (MAPK) signaling (Karia et al., 2021). The role of phosphorylation cascades during the interactions between photosynthesis and respiration, in particular, includes the participation of phytochrome and cryptochrome systems, in which the first step represents light-dependent phosphorylation of the protein moiety of phytochrome and cryptochrome (Igamberdiev et al., 2014a, 2014b; Ponnu and Hoecker, 2022).

Many reactions in photosynthetic plant cells are regulated via thioredoxin (Trx; Martí et al., 2020). In mitochondria, Trx is reduced by NADPH-dependent reductases (Dreyer and Dietz, 2018). More than a hundred mitochondrial proteins are potential targets for Trx regulation (Buchanan, 2017). Two TCA cycle enzymes, namely, succinate dehydrogenase (the flavoprotein subunit) and fumarase, exhibit a decreased activity in response to Trx-mediated reduction (Daloso et al., 2015), while citrate synthase is activated by Trx (Schmidtmann et al., 2014). Daloso et al. (2015) constructed a double mutant of Arabidopsis with down-regulated NADP-Trx reductase (*ntra* and *ntrb* genes) and Trx *o1* in mitochondria. They showed that while Trx deactivates succinate dehydrogenase and fumarase in mitochondria, it activates the cytosolic citrate synthase. This became one of the first studies demonstrating pathway-specific regulation of TCA cycle enzymes by Trx. The mitochondrial isoform of citrate synthase in Arabidopsis was also shown to be regulated by Trx (Schmidtmann et al. 2014). The authors demonstrated that oxidation inhibits mitochondrial citrate synthase activity by the formation of mixed disulfides, resulting in the accumulation of large redox-dependent aggregates. Trx can cleave diverse intramolecular and intermolecular disulfide bridges reversing the enzyme to the active state.

The Trx-dependent regulation of the TCA cycle enzymes contributes to the TCA cycle operation in a noncyclic mode in the light (Møller et al., 2020a, 2020b). Redox states of several Trxs directly follow the linear electron transport rate in photosynthesis (Zimmer et al., 2021). While the redox targets have kinetics compatible with equilibrium with one Trx, the reduction of other proteins manifests specific kinetic limitations, permitting flexible adjustment of the redox state for each component of plant metabolism (Zimmer et al., 2021). Disulfide bridge formation, which regulates the activities of many respiratory enzymes including AOX, is reversible through thioredoxin (Trx)- or glutaredoxin (Grx)-catalyzed reduction (Nietzel et al., 2017). Thioredoxin reductase (Trx-R) regenerates Trx *o* to the reduced form, and for this reaction, NADPH can be supplied by NADP-isocitrate dehydrogenase (da Fonseca-Pereira et al., 2021). Moreover, the negative regulation of the TCA cycle by the Trx system is coordinated with an increased input of electrons into the AOX pathway (Florez-Sarasa et al., 2019). Glutaredoxin GRXS15 is required for the biosynthesis of lipoyl-dependent dehydrogenases in mitochondria (Moseler et al., 2021).

All complexes of the mitochondrial ETC and AOX are regulated by the thioredoxin system (reviewed in Møller et al., 2020a, 2020b). Interaction of Trx *o2* with the activating CBS domain-containing protein CBSX3 was shown to regulate ROS generation in plant mitochondria at the level of complex II (succinate dehydrogenase; Shin et al., 2020). Redox regulation via Trx *o1* allows for the rapid initiation of mitochondrial steps of the photorespiratory cycle, which, in turn, facilitates the light-triggered induction of photosynthesis (Reinholdt et al., 2019). Trx *h2* plays an important role in the redox regulation of mitochondrial photorespiratory metabolism (da Fonseca-Pereira et al., 2020). A number of transcription factors (TFs) contain redox-sensitive cysteine residues at their DNA-binding sites, and hence, ROS-induced thiol oxidation strongly inhibits their recognition of the cognate DNA sequences, resulting in the redox regulation at the level of transcription (Munné-Bosch et al., 2013). The level of RNA editing represents another essential point of regulation, which is particularly important for the assembly of the mitochondrial ETC complexes (He et al., 2018; Maldonaldo et al., 2022) and requires further investigation.

## Conclusions and perspectives

The photosynthetic performance of plants has to be optimized to be efficient, prevail against stress-induced disturbances, and provide maximum productivity in utilizing energy to build biomass. This is achieved via establishing conditions for overcoming environmental disturbance through the autocatalytic feedbacks incorporated into the integral intercompartmental organization of the plant cell. Mitochondria are directly involved in this role in the light via regulating the noncoupled pathways of electron transport, catalyzing the exchange of malate and citrate, contributing to the balance of adenylates, NAD, and NADP in the cytosol and other compartments, and driving the operation of the cytochrome P450 monooxygenase and 2-OG-dependent dioxygenase systems. The latter systems generate a vast number of secondary metabolites and other compounds determining the specificity and metabolic uniqueness of individual plant species. In photosynthetic plant cells, mitochondria are transformed from the powerhouse organelles to the thermodynamic buffering organelles that regulate redox and energy balance in the cell and supply 2-OG and NADPH for biosynthetic reactions. Several important aspects of this transformation require further clarification (see *Outstanding Questions*) and will be the center of future investigations.

## Advances

- Mitochondria avoid overenergization of photosynthetic cells and optimize metabolic fluxes by balancing phosphorylation of adenylates and reduction of pyridine nucleotides.
- The malate valve shuttles redox equivalents from chloroplasts and optimizes NADH/NAD^+^ ratios.
- The citrate valve, driven by mitochondria, generates NADPH in the cytosol and other compartments; efflux of citrate from mitochondria is regulated by redox level and inhibition of aconitase.
- The citrate valve provides 2-OG for amino acid biosynthesis and for 2ODD reactions; NADPH produced via the citrate valve feeds monooxygenase reactions performed by cytochrome P450.
- In the light, mitochondria transform from powerhouse organelles to thermodynamic buffering organelles coordinating and optimizing anabolic and catabolic pathways within the plant cell.

## Outstanding questions

- What are the fine mechanisms of regulation of 2ODD reactions in light and darkness?
- How do the TCA cycle and 2-OG supply to 2ODDs regulate the balance of phytohormones?
- How is mitochondrial function connected with the regulation of cytochrome P450 monooxygenase reactions?
- What are the fine mechanisms that coordinate the regulation of the TCA cycle and photorespiration at the transcriptional, translational, and post-translational levels?
- How is the thioredoxin network in plant mitochondria coordinated with photosynthetic performance?
- What molecular factors and mechanisms determine the level of inhibition of the mitochondrial respiration in the light?

## Abbreviations

AOX: alternative oxidase
ETC: electron transport chain
ICDH: isocitrate dehydrogenase
NDA: internal rotenone-insensitive NADH dehydrogenase
NDB: external rotenone-insensitive NADPH and NADH dehydrogenases
NDC: internal rotenone-insensitive NADPH dehydrogenase
OAA: oxaloacetate
2-OG: 2-oxoglutarate
2ODD: 2-oxoglutarate-dependent dioxygenase
PDC: pyruvate dehydrogenase complex
TCA cycle: tricarboxylic acid cycle
